# Role of the *Plasmodium* Export Element in Trafficking Parasite Proteins to the Infected Erythrocyte

**DOI:** 10.1111/j.1600-0854.2008.00864.x

**Published:** 2009-01-07

**Authors:** Justin A Boddey, Robert L Moritz, Richard J Simpson, Alan F Cowman

**Affiliations:** 1 The Walter and Eliza Hall Institute of Medical ResearchParkville 3050, Melbourne, Australia; 2 Joint Proteomics Laboratory, Ludwig Institute for Cancer Research and The Walter and Eliza Hall Institute of Medical ResearchParkville, 3050, Melbourne, Australia

**Keywords:** acetylation, malaria, PEXEL, signal sequence, trafficking

## Abstract

The intracellular survival of *Plasmodium falciparum* within human erythrocytes is dependent on export of parasite proteins that remodel the host cell. Most exported proteins require a conserved motif (RxLxE/Q/D), termed the *Plasmodium* export element (PEXEL) or vacuolar targeting sequence (VTS), for targeting beyond the parasitophorous vacuole membrane and into the host cell; however, the precise role of this motif in export is poorly defined. We used transgenic *P. falciparum* expressing chimeric proteins to investigate the function of the PEXEL motif for export. The PEXEL constitutes a bifunctional export motif comprising a protease recognition sequence that is cleaved, in the endoplasmic reticulum, from proteins destined for export, in a PEXEL arginine- and leucine-dependent manner. Following processing, the remaining conserved PEXEL residue is required to direct the mature protein to the host cell. Furthermore, we demonstrate that N acetylation of proteins following N-terminal processing is a PEXEL-independent process that is insufficient for correct export to the host cell. This work defines the role of each residue in the PEXEL for export into the *P. falciparum*-infected erythrocyte.

Four species of *Plasmodium* cause malaria in humans; however, *Plasmodium falciparum* is responsible for the most severe. Approximately 600 million people are infected each year resulting in more than 2 million deaths (1). Central to the intracellular survival of *P. falciparum* and pathogenesis of malaria is the extensive remodelling of the host erythrocyte during the parasite asexual blood stage (reviewed in 2). Remodelling occurs in the absence of an established secretory network in the host cell and enables nutrient uptake and surface exposure of the major adhesin, *P. falciparum* erythrocyte membrane protein 1 (PfEMP1) (3–5), which mediates cytoadherence to microvascular endothelia and placental trophoblasts, and facilitates immune evasion by antigenic variation (reviewed in 6).

To remodel the infected erythrocyte, *P. falciparum* exports effector proteins from within the parasite, through the endoplasmic reticulum (ER), to the parasite membrane and across the parasitophorous vacuole and parasitophorous vacuole membrane into the host cell (7,8). In the case of PfEMP1, the protein is trafficked with Maurer's clefts, which bud from the parasitophorous vacuole membrane, to electron dense knobs underlying the erythrocyte membrane into which it is inserted (9–11). In addition, *P. falciparum* exports a variety of proteins that play a role in protein trafficking (12–15), nutrient acquisition (16), knob formation (17) and altered erythrocyte mechanical properties (14,18).

A conserved export motif termed *Plasmodium* export element (PEXEL) (19) or vacuolar translocation signal (VTS) (20) is required for export beyond the parasitophorous vacuole membrane, either to the erythrocyte cytosol (19,20) or to the hepatocyte cytosol in the parasite liver stages (21). The PEXEL consists of a pentameric sequence RxLxE/Q/D and is conserved across *Plasmodia* and present in more than 300 *P. falciparum* proteins (22). This includes proteins such as the knob-associated histidine-rich protein (KAHRP), which is required for knob formation (17) and glycophorin-binding protein 130 (GBP130) (23). Recently, the PEXEL motif was shown to be a protease cleavage site, with processing at the conserved leucine and acetylation of the new N-terminus (24).

A definitive determination of the role of each conserved PEXEL residue has not been reported, nor has the function of N acetylation in export been characterised. In this work, we use transgenic *P. falciparum* lines expressing mutant chimaeras to elucidate the function of the PEXEL in protein sorting and to characterise the potential relationship between the PEXEL, N acetylation and export. This addresses the early molecular events required for protein export to the *P. falciparum*-infected erythrocyte.

## Results

### KAHRP and GBP130 are differentially processed at the N-terminus before export in a PEXEL-dependent manner

**Figure 1:**
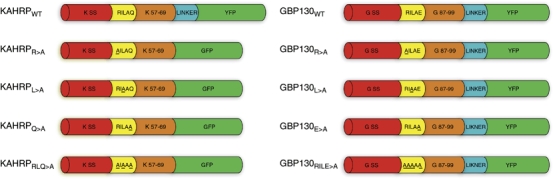
Structure of the chimaeric proteins The first 69 residues of KAHRP or 99 residues of GBP130, containing a native or mutated PEXEL,were fused to GFP or YFP. All GBP130 chimaeras and the KAHRP_WT_ chimaera were expressed from the *HSP86* promoter (19). The remaining KAHRP chimaeras were expressed from the PfCRT promoter as in-frame fusions with GFPmut2.

The PEXEL motif consists of RxLxE/Q/D and is required for protein export beyond the parasitophorous vacuole membrane (19,20). This motif is proteolytically processed during transit to the host cell (24). For consistency, the transgenic *P. falciparum* lines described previously (19) were used in the current study to analyse the N-termini of different PEXEL chimaeras during export in infected erythrocytes. The transgenic lines expressed the N-terminus of KAHRP or GBP130, with a native or mutated PEXEL, fused to green fluorescent protein (GFP) or yellow fluorescent protein (YFP) at the C-terminus (Figure 1), as previously described (19). The transgenic parasites express KAHRP_R>A_, KAHRP_L>A_ and KAHRP_RLQ>A_ as GFP chimaeras as well as KAHRP_WT_, GBP130_WT_, GBP130_R>A_, GBP130_L>A_, GBP130_E>A_ and GBP130_RILE>A_ as YFP chimaeras, which were generated by transfection of plasmids (19). Transgenic parasites expressing a KAHRP_Q>A_ GFP chimaera were generated using the vector pJABK_Q>A_Glux.1 (see *Materials and Methods*).

**Figure 2:**
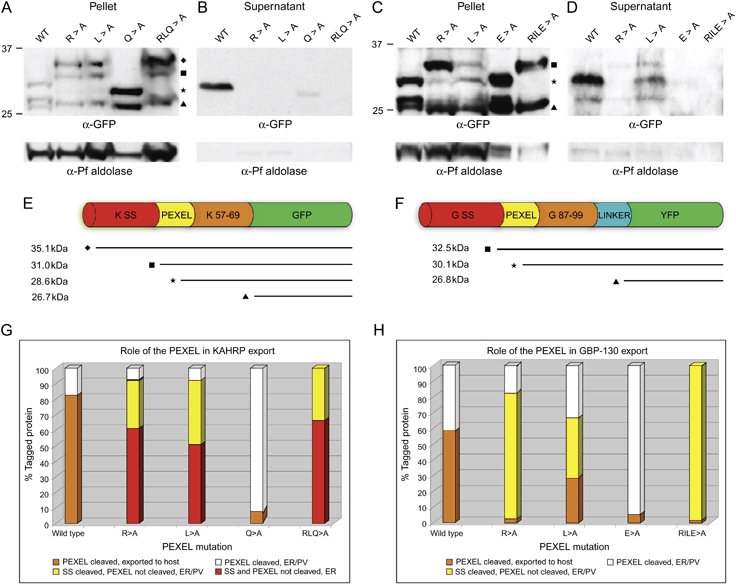
Role of the PEXEL residues in processing and export to the erythrocyte Immunoblots with α-GFP antibodies against KAHRP chimaeras from tetanolysin pellets (A) and supernatant (B) or GBP130 chimaeras from tetanolysin pellets (C) and supernatant (D) are shown. Antibodies to aldolase (48) were used as a permeabilisation control, as described previously (49) and reflects the quantity of protein loaded in each lane. While equal proportions of pellet and supernatant were loaded for each chimaera, equal loadings could not be achieved between different chimaeras because of differences in episomal expression. The densitometric analyses below (see G and H) were thus limited to comparing only between pellet and supernatant fractions of the same chimaera. Upper bands in (D, lanes 2 and 3) represent slight vacuolar leakage. E) Predicted protein sizes of KAHRP chimaeras after differential N-terminal processing; ♦ represents full-length chimaeras with signal sequence; ▪ represents processing at/near the site predicted by SignalP; H represents chimaeras processed downstream of prediction by SignalP (i.e. within the PEXEL); ▴ represents degradation to GFP/YFP only (confirmed by MS; Figure S2). The linker upstream of YFP in KAHRP_WT_ (Figure 1) is not depicted here. While the N-termini of each KAHRP chimaera are the same, except for the mutations shown, the C-terminal YFP linker in KAHRP_WT_ explains the minor size shift in (A) and (B) between WT and other chimaeras processed at the PEXEL (i.e. those depicted with *). F) Predicted protein sizes of GBP130 chimaeras after differential N-terminal processing. Varying exposures within the linear range of the blots represented in (A–D) were scanned at high resolution and densitometry was undertaken to approximately quantify the differential N-terminal processing and cellular localisation of KAHRP (G) and GBP130 (H) chimaeras. For each chimaera, percentages were calculated by dividing the intensity of each band in the supernatant (exported to host) or pellet (ER or parasitophorous vacuole) by the sum of the band intensities for that chimaera (total tagged chimaera) and multiplying by 100. Percentages between chimaerae are directly comparable. WT, wild type; SS, signal sequence; PV, parasitophorous vacuole.

To separate exported chimaeras (erythrocyte cytosol) from nonexported chimaeras (parasite and parasitophorous vacuole) and enable visualisation of potential processing differences, we used the selective pore-forming toxin tetanolysin (25) followed by analysis of the fractions by immunoblot with α-GFP antibodies. A number of different sized GFP chimaerae was observed in the tetanolysin pellets, suggesting PEXEL-dependent N-terminal processing (Figure 2A,C). No size difference was observed for chimaeras with a wild-type PEXEL between tetanolysin supernatants (exported) and pellets (not exported) (Figure 2B,D), suggesting PEXEL-dependent processing occurred before translocation to the host cell. This agrees with previous work that suggested PEXEL processing occurred in the parasite ER before export (24).

PEXEL processing differed between wild type and mutant KAHRP and GBP130 chimaerae. For KAHRP, with a wild-type PEXEL, the protein was processed to smaller species and efficiently exported (Figure 2B,G). However, for the R>A, L>A and RLQ>A PEXEL mutants, we observed two protein species (indicated by ♦ and ▪, Figure 2A) that were larger than observed for wild type and the Q>A PEXEL mutant, suggesting that the specific amino acid changes had interfered with normal processing and, consequently, export (Figure 2A). The uppermost species (♦) was approximately 35 kDa, consistent with the predicted size of the full-length KAHRP chimaera (i.e. atypical signal sequence still attached). The second species (▪) was approximately 31 kDa, consistent with processing of the atypical signal sequence at/near the site predicted by SignalP (^32^LKC-SN^36^). The 31-kDa species was clearly larger than the bands observed for wild type and Q>A PEXEL mutant proteins (Figure 2A,E), indicating that the latter chimaeras were further processed at the N-terminus. Therefore, the R>A and L>A mutation in the KAHRP PEXEL results in incorrect processing and, as a result, the chimaeras are not exported to the erythrocyte in measurable quantities (Figure 2B,G).

For GBP130, a protein species of approximately 33 kDa was detected (indicated by ▪ in Figure 2C) for the R>A, L>A and RILE>A PEXEL mutants that was larger than the wild type (indicated by H, Figure 2C) and E>A PEXEL mutant (Figure 2C,F). The 33-kDa protein was a size consistent with processing of the atypical signal sequence at/near the site predicted by SignalP (^67^ICG-DK^71^) (Figure 2F). Full-length GBP130 chimaeras with the atypical signal sequence (predicted mass 39.7 kDa) were not obviously present in contrast to the KAHRP proteins (results not shown). Furthermore, approximately 60% of the GBP130_L>A_ chimaera was processed to the same size as the GBP130_WT_ and GBP130_E>A_ chimaeras (Figure 2C, lanes 1, 3 and 4), suggesting that this mutation did not ablate PEXEL cleavage, consistent with a proportion of the cleaved protein being found in the tetanolysin supernatant (Figure 2D). The E>A mutation did not affect processing of the GBP130 PEXEL and some export of the protein did occur; however, this was much less than the nonmutated form. These results show that the conserved R and L PEXEL residues are critical for efficient proteolytic cleavage. However, the E/Q>A mutation does not affect processing but alters the level of export to the host cell.

It is noteworthy that we observed differences in episomal expression of the different constructs, as reflected by the quantity of aldolase in Figure 2A–D. It is possible that this influenced the efficiency of N-terminal processing and export to the host cell of the chimaeras.

### Exported GBP130 is processed at the PEXEL leucine and acetylated at the new N-terminus

**Figure 3:**
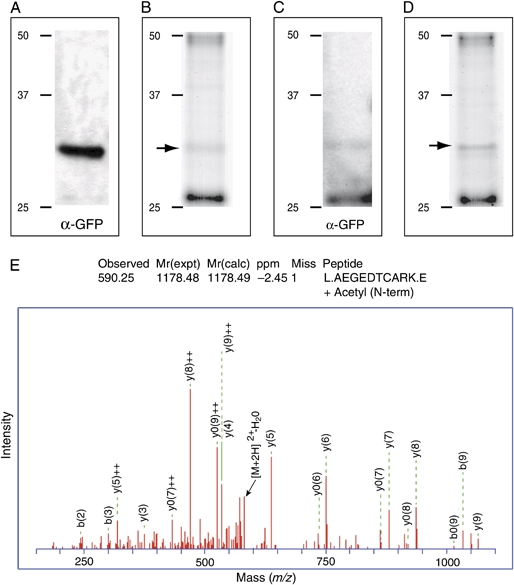
Affinity purification of exported GBP130_WT_ and KAHRP_WT_ chimaeras and MS Immunoblot(A) and coomassie gel (B) of the GBP130_WT_ chimaera after affinity purification from the saponin supernatant. Immunoblot (C) and coomassie gel (D) of the KAHRP_WT_ chimaera after affinity purification from the saponin supernatant. The bands indicated by an arrow in (B) and (D) were excised and subjected to MS. The lowest band (∼26 kDa) is degraded protein (YFP only). E) Mass spectra of the most N-terminal peptide from the band in (B) showing that exported GBP130 was processed in the PEXEL after leucine and acetylated at the new N-terminus.

To confirm the identity of the different processed chimaerae, we used affinity purification and nano-liquid chromatography tandem mass spectrometry (LC-MS/MS) to characterise the N-termini of the processed species. Transgenic *P. falciparum* lines expressing KAHRP_WT_ and GBP130_WT_ chimaeras were prepared and purified from the supernatant (exported protein) by immunoaffinity chromatography with α-GFP agarose, separated by SDS–PAGE, verified by immunoblot (Figure 3A,C) and excised from gels (Figure 3B,D). Purified chimaeras were reduced, alkylated and in-gel digested with trypsin (GBP130) or chymotrypsin (KAHRP) and subjected to LC-MS/MS to determine the mass and amino acid sequence of N-terminal peptides.

The GBP130_WT_ YFP chimaera was identified with 35% sequence coverage and the most N-terminal peptide had a mass of *m/z* 1178.49 and the sequence Ac-^87^AEGEDTCARK^96^ (Figure 3E). The nonacetylated form of this species was not detected, illustrating the homogeneous nature of the chimaera fraction. The identification of this peptide indicates that proteolytic processing of the GBP130 PEXEL occurred *in vivo*, with the PEXEL leucine at the P1 position (i.e. ^84^RIL-AE^88^), and the protein acetylated at the new N-terminus after processing, in agreement with a previous study on other proteins (24). We attempted to identify the PEXEL-containing chymotryptic peptide with sequence ±Ac-^57^AQKQHEHHHHHHHKGGRADPAF^78^ from the KAHRP_WT_ YFP chimaera; however, the configuration of the MALDI-Qstar quadrupole time-of-flight instrument did not allow us to detect this histidine-rich peptide. While we detected peptides on the C-terminal side of the histidine-rich sequence (e.g. ^113^SVSGEGEGDATYGKL^127^; results not shown), we were unable to detect peptides on the N-terminal side of the PEXEL despite the absence of histidines in that region, consistent with *in vivo* cleavage of the chimaera within the PEXEL. GBP130_WT_ was processed at the PEXEL leucine and immunoblots indicate that this occurs in a manner dependent on arginine at the P3 position and leucine at the P1 position. While we do not have definitive proteomic data on the KAHRP_WT_ cleavage, the immunodata and the peptide sequence recovered are consistent with cleavage at the leucine position of the PEXEL.

### PEXEL processing and N acetylation occur in endogenous *P. falciparum* proteins

Analysis of the total proteomic data obtained in this study revealed a number of endogenous *P. falciparum* proteins that coprecipitated with the tagged chimaeras. Within the pellet fractions, these included the five coat protein complex II (COPII) proteins and three COPI subunits (results not shown) and many exported proteins, suggesting only partial solubilisation of our samples containing vesicles enriched for proteins destined for export. The supernatant fractions contained additional exported proteins. We were able to identify the PEXEL-containing peptide from seven endogenous proteins (Figure S1), six of which were N acetylated (Table 1). Two of the proteins have lysine in place of arginine in the PEXEL (Table 1), indicating that lysine at position P3 can facilitate processing within the PEXEL. Furthermore, two proteins contained a predicted transmembrane spanning domain (Table 1) suggesting that they traffic in a similar manner as soluble proteins. Collectively, these data confirm that PEXEL processing and N acetylation occur in a number of endogenous exported proteins and validate our use of chimeric proteins to study PEXEL function.

**Table 1:** Identification of endogenous *Plasmodium falciparum* proteins by MS that were processed within the PEXEL at leucine and N acetylated

Protein	PEXEL	Most N-terminal peptide	N acetylation	TM^a^	Fraction^b^	Digest^c^
PFA0210c	RILKE	^64^KENKEESLETAAVNENTK^81^	No	0	P	T
PFD0115c	RILVE	^62^VEFSNSYYYDEPK^74^	Yes	0	S	T
PFE0050w	RVLAE	^92^AEQEDQYIR^100^	Yes	0	S	T
PFI1780w	KSLAE	^60^AEASPEEHNNL^70^	Yes	0	P	C
PF10_0020	RKLAE	^89^AEALKDDERFEK^100^	Yes	0	S	T
PF13_0275	RILTQ	^89^TQGDHHEETEDVNHK^103^	Yes	1	S	T
MAL3P8.15	KSLAE	^89^AEMDHTK^95^	Yes	1	S	T

a Number of putative transmembrane domains in the predicted protein.

b Proteins coprecipitated from the saponin pellet (P) or supernatant (S).

c Proteins were digested with trypsin (T) or chymotrypsin (C) for MS.

### PEXEL processing and N acetylation occur before export to the parasitophorous vacuole

**Figure 4:**
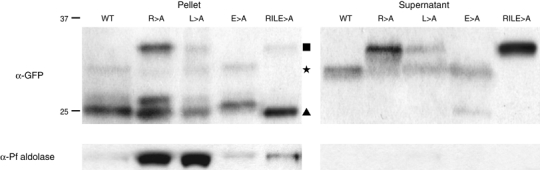
Processing within the PEXEL occurs before export to the parasitophorous vacuole Immunoblot with α-GFP antibodies against GBP130 chimaeras fractionated with saponin indicates that processing occurred in the parasite fraction (pellet) before export. Antibodies to aldolase were used to validate parasite membrane integrity following saponin treatment (note the presence of aldolase in the pellet fractions but absence from the supernatant fractions) and reflects the quantity of protein loaded in each lane. More of the GBP130_R>A_ and GBP130_L>A_ chimaera samples were loaded because of rapid loss of fluorescence over time in parasites overexpressing these mutant chimaeras. The GBP130_RILE>A_ chimaera traffics predominantly to the parasitophorous vacuole (see supernatant fraction). That unprocessed bands of GBP130_R>A_, GBP130_L>A_ and GBP130_RILE>A_ are present in the supernatant fraction confirms breakdown of the parasitophorous vacuole membrane by saponin.

As described above, we observed N-terminal processing of chimaeras present in tetanolysin pellets (nonexported proteins) (Figure 2A,C), indicating that processing occurred either within the parasite or in the parasitophorous vacuole. We further analysed the location of N-terminal processing and acetylation using saponin to fractionate chimaeras present only in the parasite (pellet) from those in the parasitophorous vacuole and erythrocyte cytosol (supernatant). Transgenic *P. falciparum*-infected erythrocytes were magnet purified, treated with 0.15% saponin and analysed by immunoblot. We observed the same profile of N-terminal processing of each chimaera in both the pellet and the supernatant (Figure 4), indicating that N-terminal processing occurred within the parasite. We purified the GBP130_WT_ YFP chimaera from the large-scale saponin pellet sample, solubilised in Triton-X-100, by immunoaffinity chromatography and subjected it to LC-MS/MS. Similar to the saponin supernatant, we identified the most N-terminal peptide at mass *m/z* 1178.49 with the sequence Ac-^87^AEGEDTCARK^96^ (results not shown), indicating that processing at PEXEL leucine and N acetylation of GBP130 occurred before export to the parasitophorous vacuole. We also observed significant amounts of GBP130_R>A_, GBP130_L>A_ and GBP130_RILE>A_ chimaeras in the saponin supernatant, indicating localisation in the parasitophorous vacuole of the pools of mutant chimaeras not processed in the PEXEL (Figure 4).

### Attenuation of KAHRP PEXEL processing affects signal sequence processing

**Figure 5:**
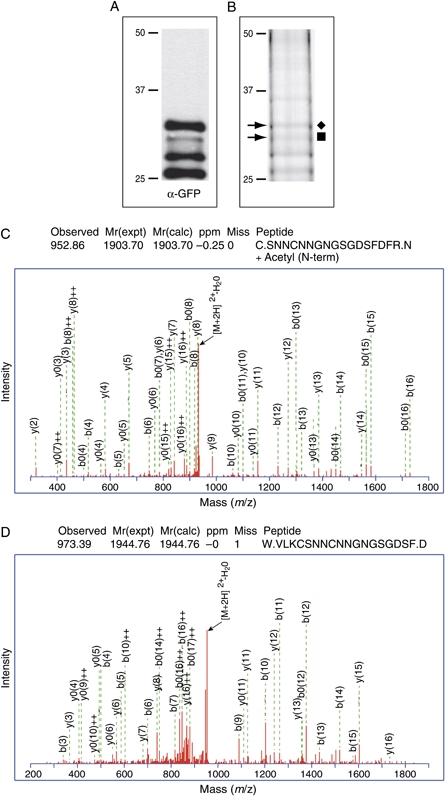
Preventing KAHRP PEXEL processing by mutation affects signal sequence processing Immunoblot(A) and coomassie gel (B) of KAHRP_R>A_ chimeric proteins after immunoaffinity purification from the saponin pellet. The bands indicated by arrows in (B) were excised and subjected to MS. C) Mass spectra of the most N-terminal peptide from the second upper band (▪) in (B) showing that the chimaera is processed at the site predicted by SignalP (^32^LKC-SNN^36^) and N acetylated. D) Mass spectra of the most N-terminal peptide from the uppermost band (♦) in (B) showing that the chimaera contains residues present in the signal sequence (i.e. N-terminal to the SignalP processing site; ^31^VLKC^34^) and has retained the signal sequence.

We sought to characterise the N-termini of the different protein populations present with respect to mutation of the KAHRP PEXEL (Figure 2A). The KAHRP_R>A_ chimeric proteins were purified by immunoaffinity chromatography from the saponin pellet (parasite located) and four GFP-containing bands were observed (Figure 5A). The upper two bands of 31 and 35 kDa were the same larger bands observed previously (Figure 2A), while the lower two bands represent some processing in the PEXEL of the large-scale sample (29 kDa; also faintly present in Figure 2A) and degradation to GFP (26.5 kDa; also present in Figure 2A), respectively, The upper two bands were excised from a gel (arrows in Figure 5B) and subjected to LC-MS/MS. From the 31-kDa protein species (▪ in Figure 5B), we identified the KAHRP_R>A_ chimaera with 83% coverage and the most N-terminal peptide had a mass of *m/z* 1903.70 with the sequence Ac-^35^SNNCNNGNGSGDSFDFR^51^ (Figure 5C), indicating that this species was processed, not within the PEXEL, but at the site predicted by SignalP (^32^LKC-SN^36^) and, notably, was N acetylated.

Furthermore, for the 35-kDa species (♦ in Figure 5B), we identified the KAHRP_R>A_ chimaera with 75% coverage and the most N-terminal peptide had a mass of *m/z* 1944.76 with the sequence ^31^VLKCSNNCNNGNGSGDSF^48^ (Figure 5D), indicating that this population of the KAHRP_R>A_ chimaera had retained the signal sequence. The presence of these protein species indicates that attenuation of processing within the KAHRP PEXEL because of a P3 arginine mutation results in processing at the signal sequence or retention of the signal sequence. The detection of N acetylation following signal processing suggests that it may be common in processed proteins of *P. falciparum*.

### KAHRP and GBP130 chimaeras localise to the ER in addition to the parasitophorous vacuole or erythrocyte cytosol

**Figure 6:**
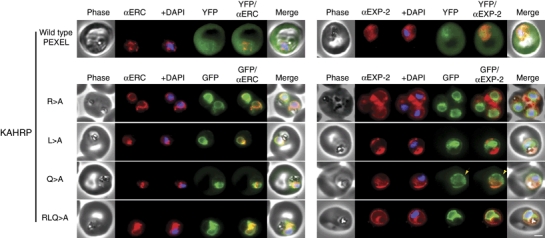
KAHRP chimaeras localise to the endoplasmic reticulum in addition to the parasitophorous vacuole or erythrocyte cytosol Images captured by immunofluorescence microscopy show substantial colocalisation of KAHRP chimaeras with the endoplasmic reticulum protein ERC (left panels). Intraparasitic fluorescence is also evident when chimaeras were colocalised with the parasitophorous vacuole membrane protein EXP-2 (right panels). Only the wild type (WT) PEXEL chimaera (uppermost panels) is efficiently exported to the erythrocyte cytosol, but low-level fluorescence in the erythrocyte cytosol was observed for the KAHRP_Q>A_ chimaera. All mutants show some accumulation within the parasitophorous vacuole but while the KAHRP_Q>A_ chimaera colocalises with ERC less than the other KAHRP mutants (left panel), it accumulates more in the parasitophorous vacuole in slightly older parasites (right panel). Accumulation of the KAHRP_Q>A_ chimaera in the parasitophorous vacuole appeared inverse to the ‘necklace of beads’ observation described previously (7; see yellow arrows in right panel). The white bar in the last panel corresponds to 2 μm for each of the panels within the figure.

**Figure 7:**
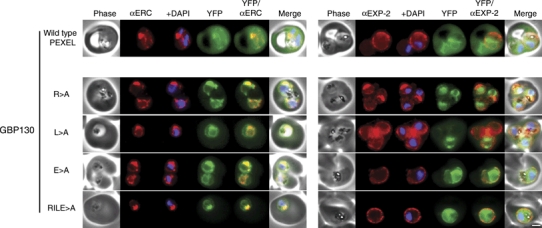
GBP130 chimaeras localise to the endoplasmic reticulum in addition to the parasitophorous vacuole or erythrocyte cytosol Images captured by immunofluorescence microscopy show substantial colocalisation of GBP130 chimaeras with the endoplasmic reticulum protein ERC (left panels). Intraparasitic fluorescence is also evident when chimaeras were colocalised with the parasitophorous vacuole membrane protein EXP-2 (right panels). Only the wild-type PEXEL chimaera is efficiently exported to the erythrocyte cytosol (uppermost panels). All mutants show some accumulation within the parasitophorous vacuole and low-level fluorescence in the erythrocyte cytosol (lower panels), and this is evident, upon careful examination, in previously published images of these chimaeras (19). The presence of GBP130_RILE>A_ in the saponin supernatant in Figure 4 suggests localisation predominantly in the parasitophorous vacuole (signal sequence processed). This is confirmed by immunofluorescence microscopy (compare GBP130_RILE>A_ in this figure to KAHRP_RLQ>A_ in Figure 6, the latter of which retains the signal sequence and localises more in the ER as a result). The white bar in the last panel corresponds to 2 μm for each of the panels within the figure.

To confirm the effect of PEXEL mutations on subcellular localisation of the KAHRP and GBP130 chimaerae, we used immunofluorescence analysis (IFA) to colocalise them with known markers of the ER and parasitophorous vacuole (Figures 6 and 7). Consistent with previous results (19), we observed accumulation of each PEXEL mutant chimaera in the parasitophorous vacuole, as shown by colocalisation with the parasitophorous vacuole membrane protein EXP-2 (Figures 6 and 7) (26). We also observed colocalisation with the endoplasmic reticulum calcium-binding protein ERC (Figures 6 and 7) (27) consistent with the ER as a transit organelle during export. We observed strong fluorescence of the KAHRP_R>A_, KAHRP_L>A_ and KAHRP_RLQ>A_ GFP chimaeras in the ER but decreased levels for the KAHRP_Q>A_ GFP chimaera (Figure 6). This may be attributed to accumulation at the ER membrane of the subspecies of each mutant chimaera that retained the signal peptide. If so, this would suggest that the proteins were retained in a folded state.

We detected low-level fluorescence in the erythrocyte cytosol of cells infected with *P. falciparum* expressing KAHRP_Q>A_ GFP and all GBP130 chimaeras; however, the intensity varied between chimaeras and was always less than that observed for the wild type chimaeras (Figures 6 and 7; refer also to Figure 2). Furthermore, we observed a pattern of accumulation in the parasitophorous vacuole, particularly for the KAHRP_Q>A_ GFP chimaera, that appeared inverse to the previously reported ‘necklace of beads’ (7), such that small foci lacking any fluorescence were observed between larger fluorescent regions (Figure 6, panel Q>A, see yellow arrows).

### N acetylation does not require a native PEXEL and is insufficient for correct export

**Figure 8:**
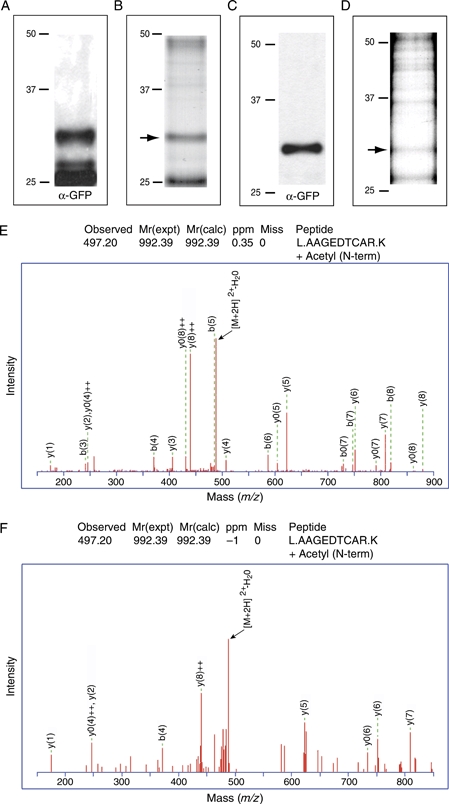
PEXEL processing and N acetylation does not require the full-length PEXEL Immunoblot (A) and coomassie gel (B) of the GBP130_E>A_ chimaera after affinity purification from the saponin pellet. Immunoblot (C) and coomassie gel (D) of the GBP130_E>A_ chimaera after affinity purification from the saponin supernatant. The bands indicated by an arrow in (B) and (D) were excised and subjected to MS. The lowest band (∼26 kDa) is degraded protein (YFP only). E) Mass spectra of the most N-terminal peptide from the band in (B) showing that GBP130_E>A_ was processed in the parasite (pellet) within the PEXEL after leucine and N acetylated. F) Mass spectra of the most N-terminal peptide from the band in (D) showing that GBP130_E>A_ in the parasitophorous vacuole (supernatant) was processed in the PEXEL after leucine and N acetylated.

To determine the requirement for N acetylation of the cleaved PEXEL and protein export, we immunoaffinity purified the GBP130_E>A_ chimaera, as it was processed identically to the GBP130_WT_ chimaera (Figure 2C, lane 4), but was poorly exported to the host cell (Figures 2D,H and 7). The chimaera was purified from both the saponin supernatant and the pellet, verified by immunoblot (Figure 8A,C) and excised from a gel (Figure 8B,D) and subjected to LC-MS/MS. We identified the GBP130_E>A_ chimaera from the saponin supernatant with 46% coverage and the most N-terminal peptide had a mass of *m/z* 992.39 with the sequence Ac-^87^AAGEDTCAR^95^ (Figure 8E). We also identified the GBP130_E>A_ chimaera from the saponin pellet with 41% coverage and the most N-terminal peptide had a mass of *m/z* 992.39 with the sequence Ac-^87^AAGEDTCAR^95^ (Figure 8F). Collectively, this indicates that processing within the PEXEL and N acetylation does not require the PEXEL glutamic acid. Furthermore, substantial accumulation of the GBP130_E>A_ chimaera in the parasitophorous vacuole indicates that processing within the PEXEL and N acetylation is insufficient for normal export, underscoring the important role played by the conserved PEXEL residue after processing.

## Discussion

Identification of the PEXEL/VTS (19,20) advanced our understanding of *P. falciparum*-host interactions as it allowed the prediction of more than 300 proteins that constitute the ‘exportome’ (22). While some studies have characterised the function of exported proteins that contain a PEXEL (14,21), little is known about the machinery involved in their trafficking pathway or how each PEXEL residue contributes to export. The PEXEL was recently identified as a recognition sequence for protease processing with subsequent N acetylation of the cleaved protein; however, the function of the conserved amino acids in this motif has not been addressed (24) nor has the function of N acetylation in export been characterised. In this work, we have determined the role of each conserved amino acid within the PEXEL with respect to their requirement for protease processing, N acetylation and export to the infected erythrocyte.

Using subcellular fractionation, we have shown that N-terminal processing of chimaeras occurs at the conserved leucine in a PEXEL-dependent manner, within the parasite, rather than the parasitophorous vacuole or during translocation to the erythrocyte cytosol. Identification of PEXEL cleavage at the conserved leucine is in agreement with a recent study (24). Their work also showed that brefeldin A (BFA), which inhibits PfSec7 in ER-to-Golgi transport (28), does not disrupt PEXEL processing and concluded that this event occurs early in the trafficking pathway within the ER. However, it is possible that treatment with BFA prevented the anterograde transport of the PEXEL protease in those experiments. Using different methodology, we come to a similar conclusion that suggests PEXEL processing occurs within the ER. Therefore, the fate of proteins destined for export is decided early, as previously hypothesised (29) and involves proteolytic cleavage in a manner dependent on the PEXEL arginine and leucine residues.

It was also shown that N acetylation occurs following PEXEL processing, and it was suggested that this could provide a mechanism to distinguish proteins that are destined to be exported (24). A model was proposed where the cleaved PEXEL (xE/Q/D) was required for N acetylation (24). Our analysis shows that after cleavage, either at the PEXEL motif or of the signal peptide, N acetylation occurs suggesting that it is common in *P. falciparum*. Analysis of the GBP130_E>A_ chimaera showed that the PEXEL was still cleaved after leucine and N acetylated; however, it was not exported but was trafficked by the default pathway to the parasitophorous vacuole, as shown previously for other GFP reporters that lack a PEXEL (30). This demonstrates that the PEXEL glutamic acid is not required for cleavage at the P1 leucine and that it is dispensable for N acetylation. Furthermore, accumulation of the GBP130_E>A_ chimaera in the parasitophorous vacuole indicates that although PEXEL processing and N acetylation of exported proteins occur both events are insufficient for normal export. This shows that xE/Q/D remaining after PEXEL cleavage plays an important role in export. We also identified an endogenous *P. falciparum* protein that was cleaved in the PEXEL at leucine but was not N acetylated. This protein was recovered from the saponin pellet, and thus, we cannot confirm that it was exported; however, this does represent the first example of a protein containing a native PEXEL that was cleaved at the P1 leucine but not N acetylated.

The consequence of PEXEL mutations in reporter proteins has been determined by microscopy to show that the conserved residues are required for efficient export to the infected erythrocyte (19,^20^,31). We have extended this analysis by determining the effect of mutations on cleavage of the PEXEL as well as export. Mutation of leucine within the PEXEL interferes with processing and consequently blocks export to the erythrocyte. Moreover, chimaeras containing a mutation of arginine in the PEXEL had a similar processing profile to the leucine mutants; thus, processing at the P1 leucine is also dependent on arginine at position P3. This is consistent with the strong conservation of these two residues in all the exported proteins that have been defined (19,^20^,22). It is noteworthy that we also identified two endogenous proteins that were processed at the PEXEL leucine that contained lysine in place of arginine at position P3. This indicates that lysine is suitable for processing of those proteins and validates the presence of KxLxE/Q/D proteins in the predicted exportome (22). It is interesting that the leucine to alanine mutation in both GBP130 and KAHRP still allowed some cleavage, albeit inefficient, and as a consequence, some export was observed. This is perhaps not surprising given the similarity in structure of alanine compared with leucine, as both are nonpolar, aliphatic, hydrophobic molecules often buried in folded proteins. Significantly, when all three conserved PEXEL residues were mutated, cleavage, and consequently, export, was inhibited entirely.

A substantial proportion of the chimeric protein pools were present in the ER, as shown by colocalisation with ERC. We believe that GFP derived from cleaved chimaeras should account for little of this colocalisation, as degradation of reporter proteins to GFP alone is thought to occur in the food vacuole (30). It is likely that this fluorescence represents transit through the ER of the chimeric proteins that are exported, or which traffic to the parasitophorous vacuole, and examples of this include the KAHRP_wt_, KAHRP_Q>A_ and GBP130_E>A_ chimaeras and a truncated STEVOR-GFP chimaera described recently (32). In contrast, the KAHRP_R>A_, KAHRP_L>A_ and KAHRP_RLQ>A_ pools that have an intact signal sequence appear to have been retained in the ER membrane, explaining the high level of fluorescence observed there. It was surprising to identify by MS a subpopulation of the KAHRP_R>A_ chimaera that had retained the signal sequence, and we infer that similar subpopulations of the KAHRP_L>A_ and KAHRP_RLQ>A_ also retained the signal sequence, the latter of which is of particular interest as there is no PEXEL remaining. One possibility to explain this is that the signal peptidase complex is involved in cleaving the PEXEL, as has been recently suggested (24). It is also possible that the PEXEL protease resides in close proximity to the signal peptidase, or even segregates/cleaves this motif beforehand, and that a combination of overexpression and inhibition of PEXEL processing because of mutation is associated with signal peptide retention. Interestingly, we did not specifically observe GBP130 with a signal sequence retained, and a significant proportion of mutant GBP130 chimaeras (particularly RILE>A) were observed in the saponin supernatant, suggesting localisation in the parasitophorous vacuole. Immunofluorescence microscopy also confirmed less GBP130_RILE>A_ in the ER compared with KAHRP_RLQ>A_ and this is likely to be because of the retention of the signal sequence for the latter chimaera. These differences between GBP130 and KAHRP chimaeras may be because of a number of factors, including different temporal expression (Figure 1), possible differences in episomal expression (Figure 2), the length of the atypical signal sequence of GBP130, which is 35 residues longer than that of KAHRP, or the distance between the predicted SignalP processing site and the PEXEL, which is 5 residues shorter for GBP130 than KAHRP. Although the exact nature of the PEXEL protease is unknown all the current data support the notion that cleavage occurs in the parasite ER (24). We consider it unlikely that our PEXEL chimaeras docked at the external leaflet of the ER membrane (29), as this would require processing of the PEXEL, which is BFA insensitive (24), on the cytoplasmic side of the ER membrane and this would release soluble proteins into the cytoplasm. Furthermore, we detected processing of the signal sequence of KAHRP and GBP130 when PEXEL processing was inhibited, indicating that these mutant chimaeras had access to the ER lumen.

Blockage of PEXEL processing in the KAHRP chimaera allowed determination of the signal peptidase cleavage site, which was identical to that predicted by SignalP (33). This is the first confirmation of the signal sequence processing site in KAHRP, and of any *P. falciparum* protein, and indicates that signal peptidase specificity is conserved with other eukaryotes. Additionally, processed KAHRP was acetylated at the new N-terminus following signal sequence cleavage, a feature that was also found in other N-terminal peptides in our proteomic analysis. The role of N acetylation following signal sequence processing in *P. falciparum* is unknown; however, as the KAHRP_R>A_ chimaera did not export correctly, it by itself is insufficient for export to the erythrocyte.

We have used soluble exported proteins as models in this study. There are a class of exported proteins that have a signal sequence, PEXEL and up to two putative transmembrane regions that would be inserted into a membrane in the parasite-infected erythrocyte (31). In the proteomic analysis, we have identified proteins with putative transmembrane spanning regions that are cleaved after leucine within the PEXEL. It is likely that they are trafficked in a similar manner as soluble proteins, either as soluble complexes or through the ER membrane to the vesicular machinery.

**Figure 9:**
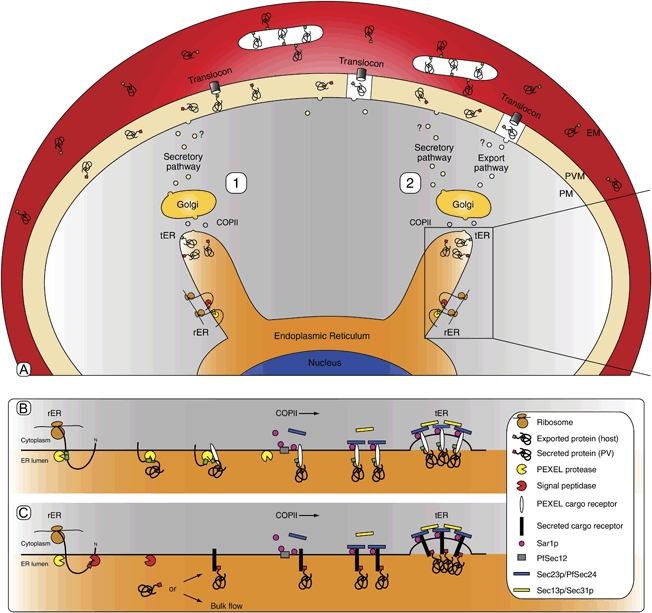
Role of the PEXEL in export of *Plasmodium falciparum* proteins to the infected erythrocyte Two proposedmodels of PEXEL-mediated export are shown (A, 1 and 2). A1) After cotranslational insertion through Sec61 at the rough endoplasmic reticulum (rER) using the signal sequence, proteins to be exported are either processed by signal peptidase (red pac-man) or sequestered and/or processed by the PEXEL protease (yellow pac-man) and sorted at the transitional endoplasmic reticulum (tER) for transport through the Golgi to the parasitophorous vacuole by the default secretory pathway. There, proteins to be exported (xE/Q/D after PEXEL processing; green protein), are recognised and trafficked across the parasitophorous vacuole membrane by a translocon. Secreted or mutated PEXEL proteins are depicted as red proteins. A2) After entry at the rER and sequestration and/or processing by either signal peptidase (red pac-man) or the PEXEL protease (yellow pac-man), proteins to be exported are differentially sorted either at the tER or at the Golgi into vesicles. This may occur through a specific transmembrane PEXEL cargo receptor that enriches functionally distinct vesicles for exported proteins (green proteins), which are targeted to subcompartments (the ‘necklace of beads’; depicted as white compartments in the parasitophorous vacuole) of the parasitophorous vacuole that houses the translocon. Exported transmembrane proteins then presumably diffuse laterally from the translocon and traffic with forming Maurer's clefts (white structures in the erythrocyte). Secreted proteins (red) traffic through the default secretory pathway to alternative compartments of the parasitophorous vacuole that do not contain the translocon. The default pathway may involve bulk flow, depicted as free red proteins in the ER that ‘sample’ the budding membrane. Uncharacterised vesicles are depicted by ‘?’. Close up (box) of the possible sorting mechanism is shown in (B) and (C). B) After cotranslational insertion into the rER PEXEL proteins are sequestered/processed by the PEXEL protease and sorted into vesicles through a transmembrane cargo receptor that interacts with the COPII machinery. C) Secreted proteins are not recognised by the PEXEL receptor but bind either a different receptor or traffic through bulk flow. The role of the Golgi is unclear but similar sorting may occur there.

While cleavage of the PEXEL motif at leucine, revealing xE/Q/D at the resulting N-terminus, is a requirement for export to the erythrocyte, the route and machinery involved are unknown. Endomembrane protein transport involves trafficking through protein-coated vesicles to target membranes. Polymerisation of COPII proteins at the transitional ER generates vesicles for transport from the ER and the heptameric COPI complex, along with ARF1, generates vesicles for reterograde transport (reviewed in 34). To achieve sorting, transmembrane cargo can interact directly with coat proteins; however, soluble cargo requires transmembrane receptors (reviewed in 35). Alternatively, proteins may traffic indirectly by bulk flow (36). *Plasmodium falciparum* contains all the COPII subunits (37–41) and at least four COPI subunits (alpha, beta, delta and epsilon) (42,43); however, the exact nature of vesicles that fuse with the parasite membrane is unknown. There are two likely pathways for export of proteins from the ER to the parasite-infected erythrocyte (Figure 9). In the first model, proteins to be exported are cotranslationally inserted into the ER membrane using the signal sequence, allowing cleavage by signal peptidase and/or recognition of the PEXEL by a specific protease that cleaves this motif. This reveals xE/Q/D at the N-terminus of the protein, which is trafficked to the Golgi and parasitophorous vacuole through the general secretory pathway using the COPII machinery. Once released into the parasitophorous vacuole a translocon, that has been hypothesised previously (2,^19^,^31^,44), would recognise the cleaved motif for export to the erythrocyte. While this model is possible, it would seem less likely as some proteins that are not exported, but may be secreted into the parasitophorous vacuole, would have xE/Q/D revealed after cleavage of the signal sequence (Boddey and Cowman, unpublished), and these mature proteins would be indistinguishable from those destined for export.

In the second model, proteins with xE/Q/D at the N-terminus after PEXEL cleavage in the ER may be sorted by a specific transmembrane PEXEL cargo receptor (soluble PEXEL proteins) or through interaction of the cytoplasmic domain (PEXEL proteins with transmembrane domain) into vesicles by the COPII machinery (Figure 9). Because an exported chimaera was recently shown to traffic through the Golgi (32), it is also possible that sorting occurs there but this again would rely on a minimal sorting sequence after PEXEL cleavage (xE/Q/D). It is unknown if N acetylation combined with xE/Q/D provides the sorting information. Vesicles with cargo to be exported rather than secreted would be functionally differentiated (29) by vesicle soluble N-ethylmaleimide-sensitive fusion protein attachment protein receptors (v-SNAREs) and would bind to the cognate target SNARE at specific domains at the parasite membrane, releasing the cargo into the parasitophorous vacuole. It has previously been reported that PEXEL-containing chimaeras within the parasitophorous vacuole have the appearance of a necklace of beads that are resistant to recovery after photobleaching, suggesting the presence of subcompartments within this space (7,45). These compartments may house a translocon that identifies and translocates proteins trafficked there. Thus, nonexported proteins would traffic through the default secretory pathway, which may involve bulk flow, to compartments in the parasitophorous vacuole that lack the translocon.

There are clearly many gaps in our understanding of the intricacies of the export pathway in *P. falciparum*. However, this work describes the initial molecular events required for effector protein export and, in defining the role of the PEXEL, significantly enhances our understanding of the export cascade in *P. falciparum*-infected cells. With malarial resistance to current therapies increasing worldwide, this export pathway represents an excellent, novel target for the design of new antimalarial drugs.

## Materials and Methods

### Parasites, plasmid constructs and transfection

*Plasmodium falciparum* strain 3D7 and transgenic parasites were cultured in human O+ erythrocytes at 4% haematocrit in RPMI 1640 medium supplemented with 25 mmHEPES pH 7.4, 0.2% sodium bicarbonate and 0.5% Albumax II (Invitrogen) in an atmosphere consisting of 5% CO_2_, 5% O_2_ and 90% N at 37°C. For consistency, we used the C-terminal GFP- and YFP-tagged chimaeras reported previously (19) to study the modifications at the N-termini of the PEXEL chimaeras when expressed in *P. falciparum*. Transgenic parasites expressing KAHRP_R>A_-, KAHRP_L>A_- and KAHRP_RLQ>A_-GFP chimaeras were generated previously (19) and thawed from liquid nitrogen. Transgenic parasites expressing KAHRP_WT_-, GBP130_WT_-, GBP130_R>A_-, GBP130_L>A_-, GBP130_E>A_- and GBP130_RILE>A_-YFP chimaeras were generated by transfection of plasmids constructed previously (19). Transgenic parasites expressing the KAHRP_Q>A_-GFP chimaera contained the construct pJABK_Q>A_Glux.1, which was made by amplifying the N-terminal sequence of KAHRP (PFB0100c) from 3D7 genomic DNA using the oligonucleotides JB102, 5′-gatctcgagATGAAAAGTTTTAAGAACAAAAATACTTTGAGG-3′ and JB103, 5′-gatcccgggATGGTGATGGTGGTGATGGTGTTCATGTTGCTTTGCTGC-3′, which introduced a Q58 to A point mutation. The amplicon was cloned into pGlux.1 using *Xho* I and *Xma* I (underlined) generating pJABK_Q>A_Glux.1. All plasmids (100 μg) were transfected into 3D7 as previously described (46) and selected with 5 nmWR99210. Trophozoite-infected erythrocytes grown to 5–10% parasitaemia were purified from uninfected cells by magnet separation through CS columns and Vario Macs magnet (Miltenyi Biotech).

### Sample fixation and immunofluorescence microscopy

Erythrocytes infected with transgenic parasites were fixed in 4% paraformaldehyde, 0.01% glutaraldehyde (Electron Microscopy Sciences) for 30 min to maintain GFP fluorescence and permeabilised in 0.1% Triton-X-100 in PBS for 10 min, all at room temperature. Cells were incubated with rabbit α-Pf ERC (1:500) or mouse α-Pf EXP-2 (1:500) for 2 h followed by Alexa Fluor 594-conjugated secondary immunoglobulin G antibodies (1:1000; Molecular Probes) for 1 h and nuclei were labelled with 4′-6-Diamidino-2-phenylindole (DAPI) nuclear stain (Roche) at 0.2 μg/mL in Vectorshield (Vector Labs). Cells were viewed with a Carl Zeiss Axioskop 2 microscope (Thornwood) and images collected using a PCO SensiCam (Motion Engineering Co.) and Axiovision 3software (Carl Zeiss). Images were assembled with Photoshop CS2 v9.0.2 (Adobe).

### Subcellular fractionation, SDS–PAGE, immunoblot and densitometry

For tetanolysin fractionation, 2 × 10^8^ infected erythrocytes were gently mixed in a solution containing 100 U/mL tetanolysin (Sigma), 0.2% bovine serum albumin (BSA; Sigma), 1× complete protease inhibitor cocktail (Roche) in PBS and incubated for 20 min at 37°C. Cells were separated into supernatant and pellet and the pellet was washed with PBS. For saponin fractionation, 2 × 10^8^ infected erythrocytes were gently mixed in a solution containing 0.09% saponin (Sigma) 0.2% BSA, 1× complete protease inhibitor cocktail in PBS for 10 min on ice. Cells were separated into supernatant and pellet and pellets were washed with PBS. All fractions were stored at −20°C. Samples were resuspended in 4× reducing SDS–PAGE sample buffer, boiled and subjected to SDS–PAGE in 10% Bis/Tris precast polyacrylamide gels (Invitrogen) and transferred to nitrocellulose membrane using an iBlot (Invitrogen). Membranes were blocked for 1 h in 5% skim milk (Diploma) in PBS containing 0.1% Tween-20 (Sigma). Membranes were probed with mouse α-GFP antibody (Roche; 1:1000) or rabbit α-Pf aldolase (1:1000) followed by horseradish peroxidase-conjugated secondary antibodies (Silenius; 1:2000) and visualised using enhanced chemiluminescence (Amersham). Densitometry of varying exposures within the linear range of blots scanned at 400 dpi was undertaken using Quantity One v4.6.3software (Bio-Rad). Comparisons were made only between equivalent loadings of pellet and supernatant fractions of each chimaera, not between different chimaeras. Band intensities were converted to proportions of whole tagged protein to account for potential errors arising from the Schwarzchild effect and to allow direct comparisons between different chimaeras.

### Chimaera preparation and purification by immunoaffinity chromotography

Following synchronisation with 1% sorbitol treatment, 1 L of transgenic parasites (per chimaera) were grown at 4% haematocrit in the presence of 20–40 nmWR99210 selection to >10% parasitaemia of trophozoites and purified by large-scale magnet separation through D columns and Super Macs magenet (Miltenyi biotech). Infected cells were fractionated with 0.15% saponin containing 1× complete protease inhibitor cocktail in PBS for 10 min on ice, separated into supernatant and pellet, and pellets were washed with PBS and all fractions were snap frozen on dry ice and stored at −80°C. Tagged chimaeras were purified directly form the saponin supernatant by mixing with 200 μL of α-GFP agarose (MBL) at 4°C for 2 h. Tagged chimaeras were solubilised from the saponin pellet in 10 pellet volumes of 1% Triton-X-100 in PBS containing 1× complete protease inhibitor cocktail and purified with α-GFP agarose, as above. Beads were thoroughly washed with PBS and eluted in 300 μL 150 mmTris pH6.8, 2 mmethylenediaminetetraacetic acid, 0.5% SDS for 10 min at room temperature, trichloroacetic acid precipitated, resuspended in SDS–PAGE sample buffer, separated by SDS–PAGE and either immunoblotted with α-GFP antibodies or stained in Imperial Protein stain (Pearce).

### Gel excision and digestion

For each sample, gel bands (∼2 mm) were excised from the 1-D gel lane and subjected to automated in-gel reduction, alkylation and tryptic digestion using the MassPREP Station (Micromass). Briefly, gel sections were automatically reduced with 10 mmDTT (Merck) for 30 min, alkylated for 20 min with 25 mmiodoacetic acid (Fluka) and digested with 150 ng trypsin (Worthington) or chymotrypsin (Roche Biochemicals) for 4.5 h at 37°C. Extracted peptide solutions (0.1% formic acid) were concentrated to approximately 10 μL by centrifugal lyophilisation using a SpeedVac AES 1010 (Savant) for nano-LC in conjunction with collisional tandem mass spectrometry (nano-LC-MS/MS).

### Nano-LC-MS/MS

A 96-well plate containing extracted peptides was placed into the micro autosampler for injection and fractionation by nanoflow reverse-phase LC on a nano-LC system (1200 series, Agilent) using a nanoAcquity C18 150 × 0.15 mm ID column (Waters) developed with a linear 60-min gradient with a flow rate of 0.5 μL/min at 45°C from 100% solvent A (0.1% formic acid in Milli-Q water) to 100% solvent B [0.1% formic acid, 60% acetonitrile (Mallinckrodt) 40% Milli-Q water]. The nano-high-performance LC was coupled on-line to an LTQ-Orbitrap mass spectrometer equipped with a nanoelectrospray ion source (Thermo Fisher Scientific) for automated MS/MS. The LTQ-Orbitrap was operated in positive ion mode for data-dependent acquisition. Survey MS scans were acquired with the resolution set to a value of 30 000. Each scan was recalibrated in real time by co-injecting of protonated polydimethylcyclosiloxane as an internal standard from ambient air into the C-trap. Up to five most intense ions per cycle were fragmented and analysed in the linear trap, with target ions already selected for MS/MS being dynamically excluded for 3 min.

### Mass spectra database searching, protein identification and bioinformatic analysis

Mass spectra peak lists were extracted using *extract-msn* as part of Bioworks 3.3.1 (Thermo Fisher Scientific) linked into Mascot Daemon (Matrix Science). The parameters used to generate the peak lists for the Orbitrap were as follows: minimum mass 400, maximum mass 5000, grouping tolerance 0.01 Da, intermediate scans 1, minimum group count 1 and 10 peaks minimum and total ion current of 100. Peak lists for each nano-LC-MS/MS run were used to search MASCOT v2.2.04 search algorithm (Matrix Science) provided by the Australian Proteomics Computational Facility (www.apcf.edu.au). Automatic charge state recognition was used because of the high-resolution survey scan (30 000). LC-MS/MS files were searched against a human, mouse, bovine and *P. falciparum* database containing the PEXEL constructs and also including a small contaminant database (keratins, trypsin, chymotrypsin, etc.). The search parameters consisted of carboxymethylation of cysteine as a fixed modification (+58 Da) with variable modifications set for NH_2_-terminal acetylation (+42 Da) and oxidation of methionine (+16 Da). A peptide mass tolerance of ±20 p.p.m., #13C defined as 1, fragment ion mass tolerance of ±0.8 Da and an allowance for up to three missed tryptic cleavages for Orbitrap data.

The signal sequence processing site of KAHRP was predicted by SignalP 3.0 (http://www.cbs.dtu.dk/services/SignalP/) with both neural networks and hidden Markov models trained on eukaryotes (33). The signal sequence processing site of GBP130 was predicted as above with SignalP 1.1 (http://www.cbs.dtu.dk/services/SignalP-1.1/) (47).

## Supplementary Information

**Figure S1: Mass spectra from seven endogenous *Plasmodium falciparum* proteins processed in the PEXEL.** Endogenous proteins that coprecipitated with the tagged PEXEL chimaeras were identified by MS. The most N-terminal peptide from seven endogenous proteins that were processed at the PEXEL leucine were identified and six such peptides were N acetylated. The mass spectra for each peptide are shown and correspond to the following proteins: A) PFA0210c, B) PFD0115c, C) PFE0050w, D) PFI1780w, E) PF10_0020, F) PF13_0275 and G) MAL3P8.15.

**Figure S2: Tagged chimaeras are degraded back to GFP.** Immunoblot (A) and coomassie gel (B) of KAHRP_R>A_ chimeric proteins after immunoaffinity purification from the saponin pellet. The band indicated by an arrow in (B) was excised and subjected to MS. C and D) Mass spectra of the two most N-terminal peptides identified from the smallest band (s) in (B) shows that the chimaera is degraded back to GFP (^72^SK-GEEL^77^ and ^77^LF-TGV^81^). Seventy-two per cent sequence coverage of the full-length chimaera (94% of the GFP sequence) was obtained.
