# Supplementary figures and images for: Role of the Plasmodium Export Element in Trafficking Parasite Proteins to the Infected Erythrocyte
Source: Traffic. 2009 Jan 7;10(3):285–99. doi: 10.1111/j.1600-0854.2008.00864.x (PMC2682620; doi:10.1111/j.1600-0854.2008.00864.x)

**A**

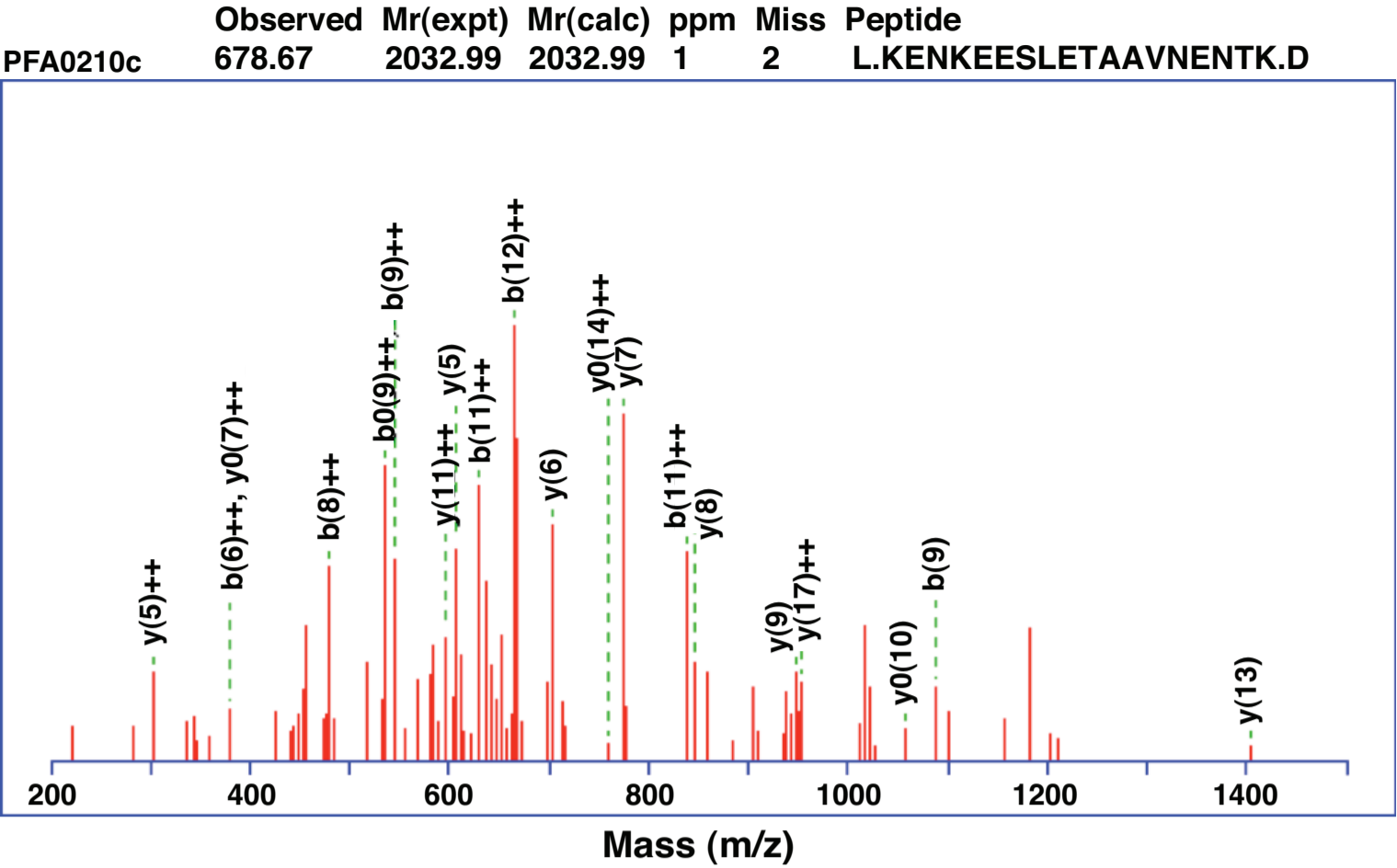

**B**

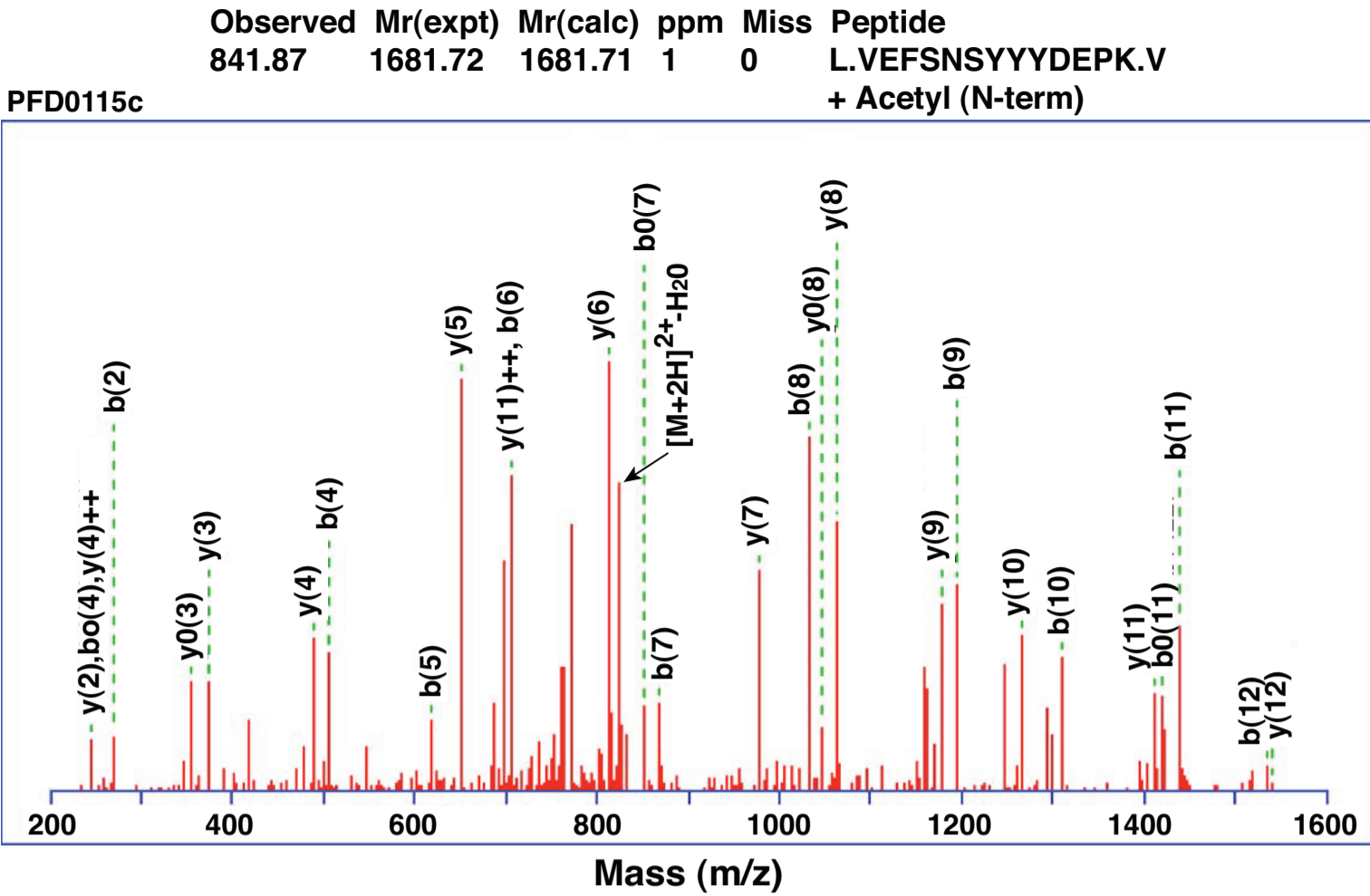

**C**

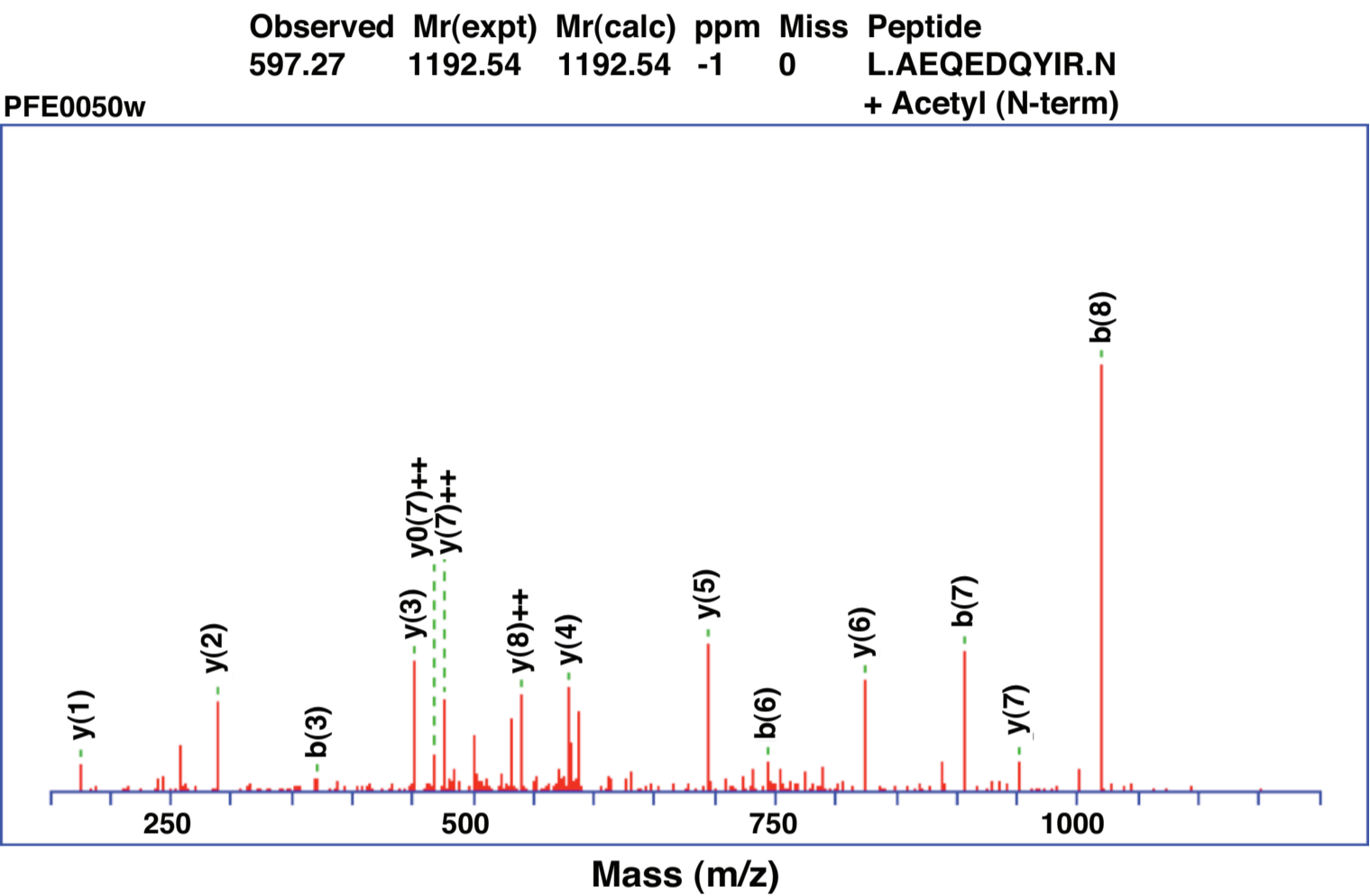

**D**

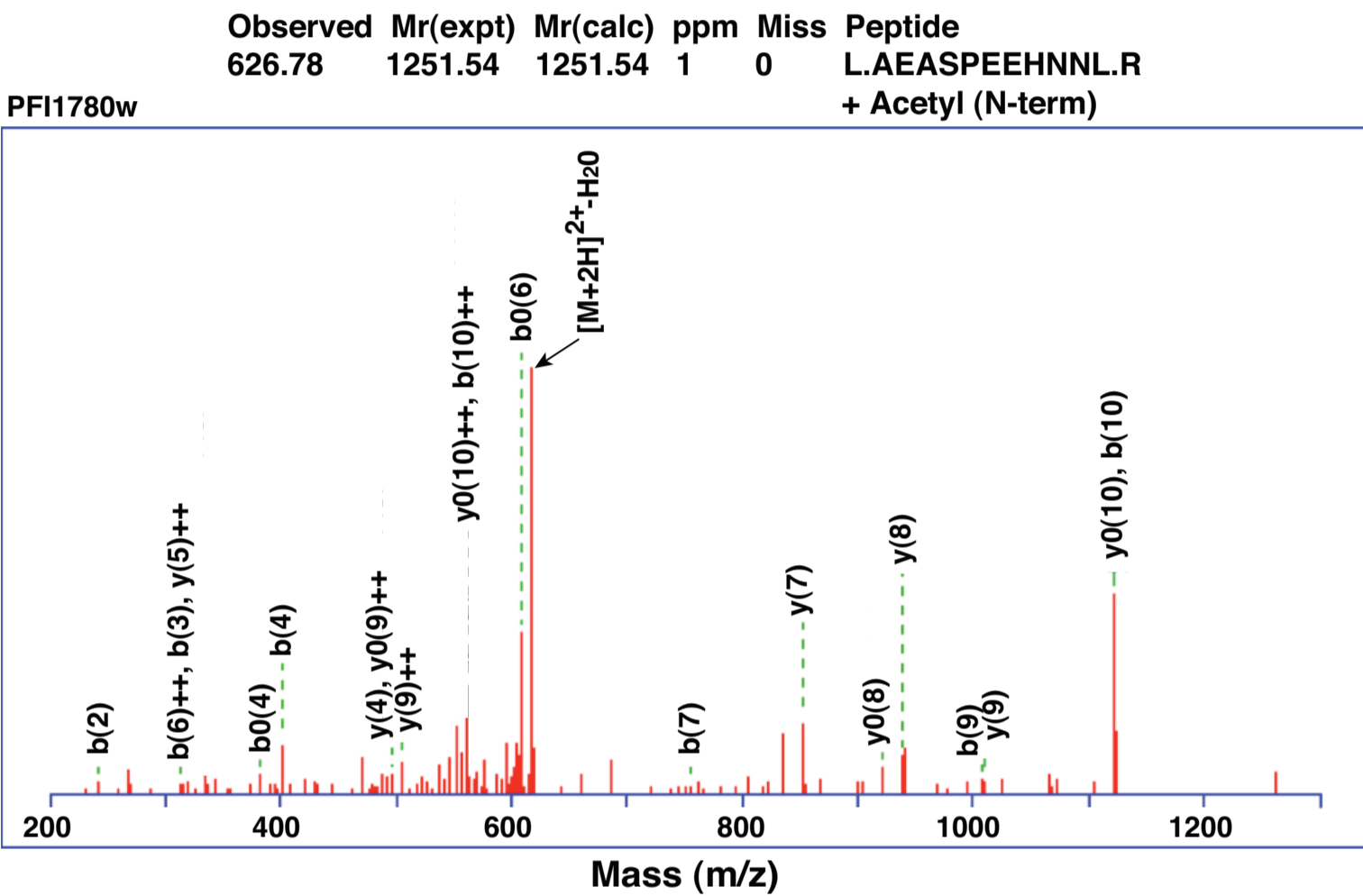

**E**

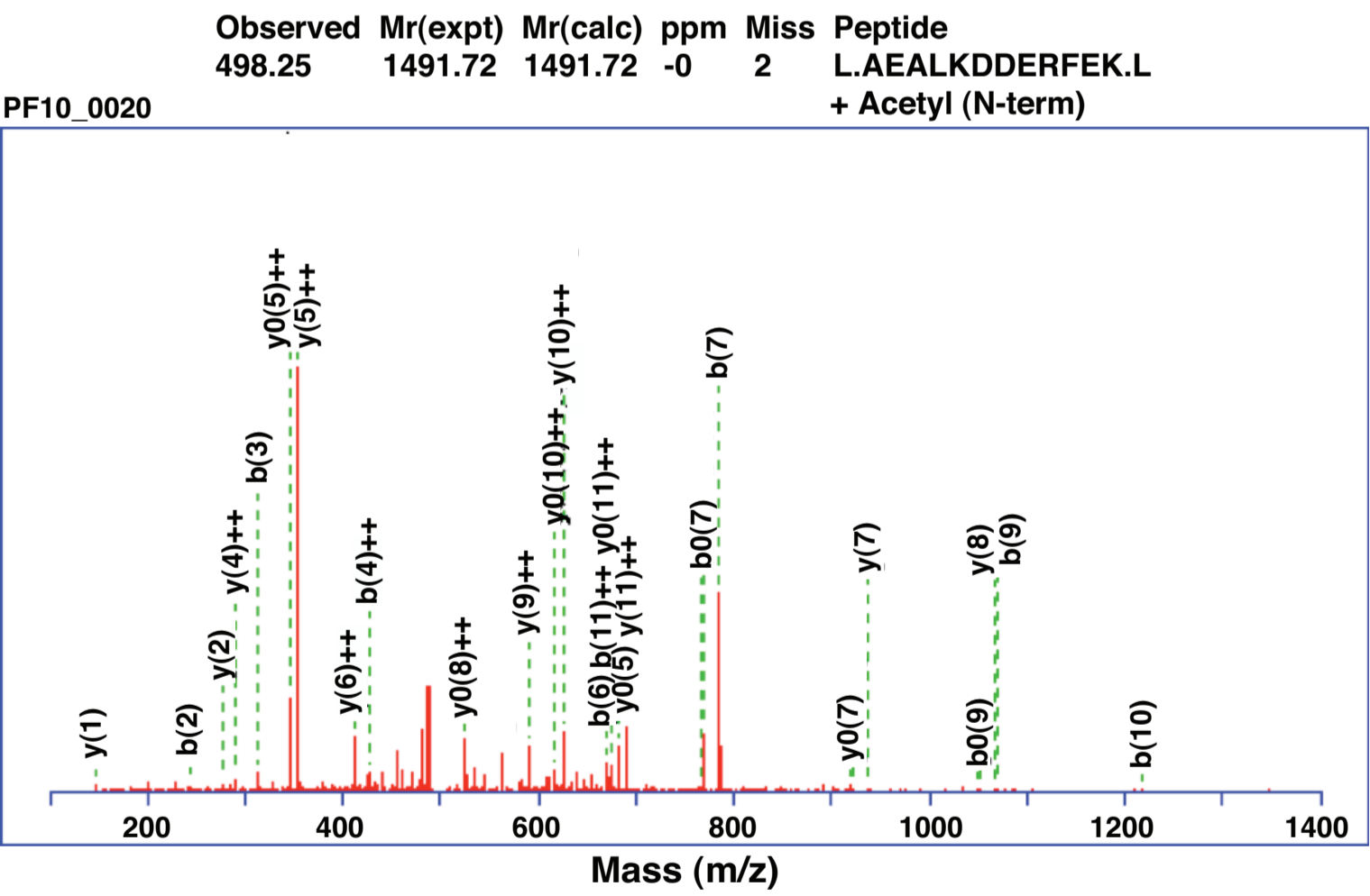

**F**

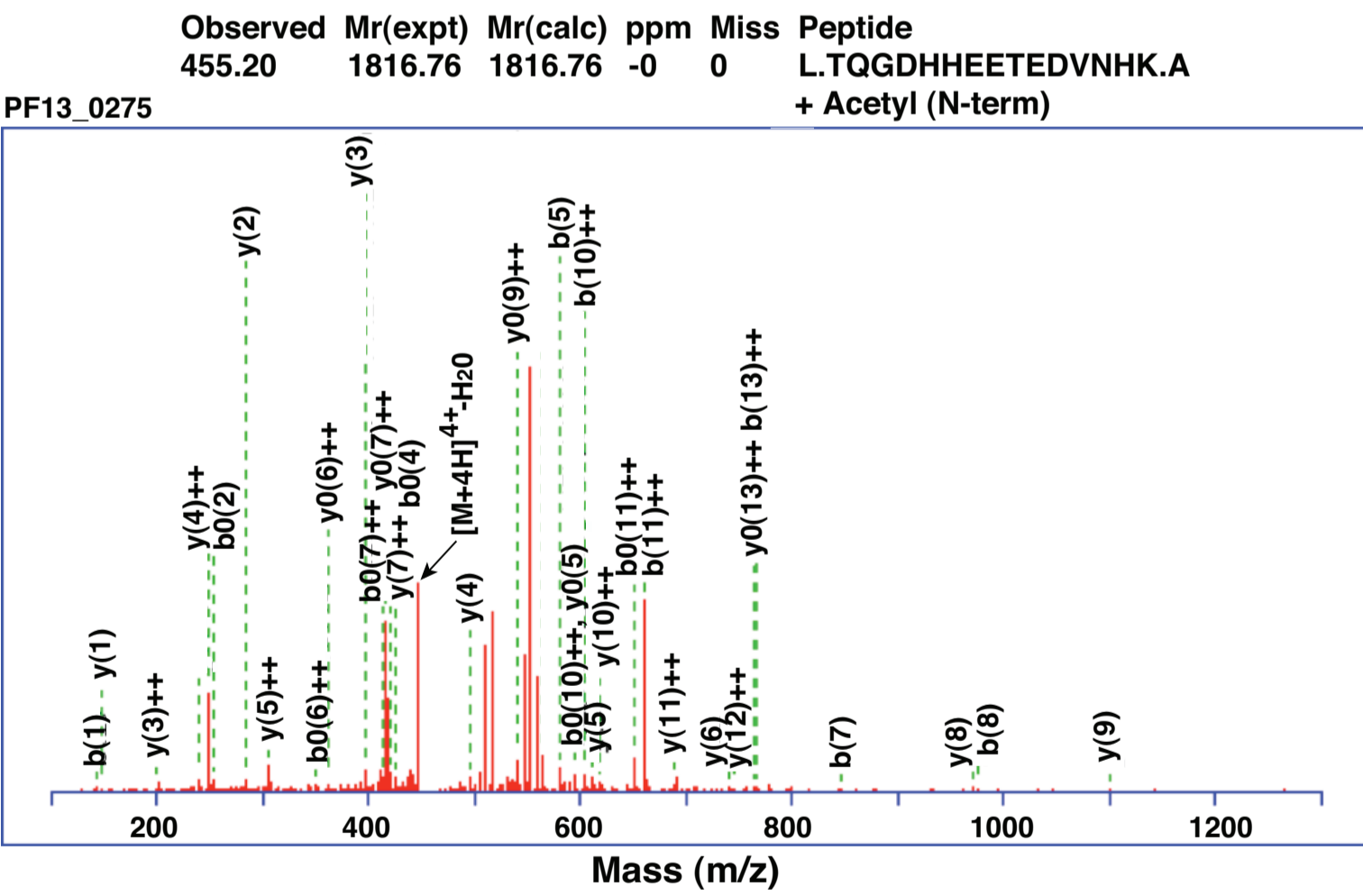

**G**

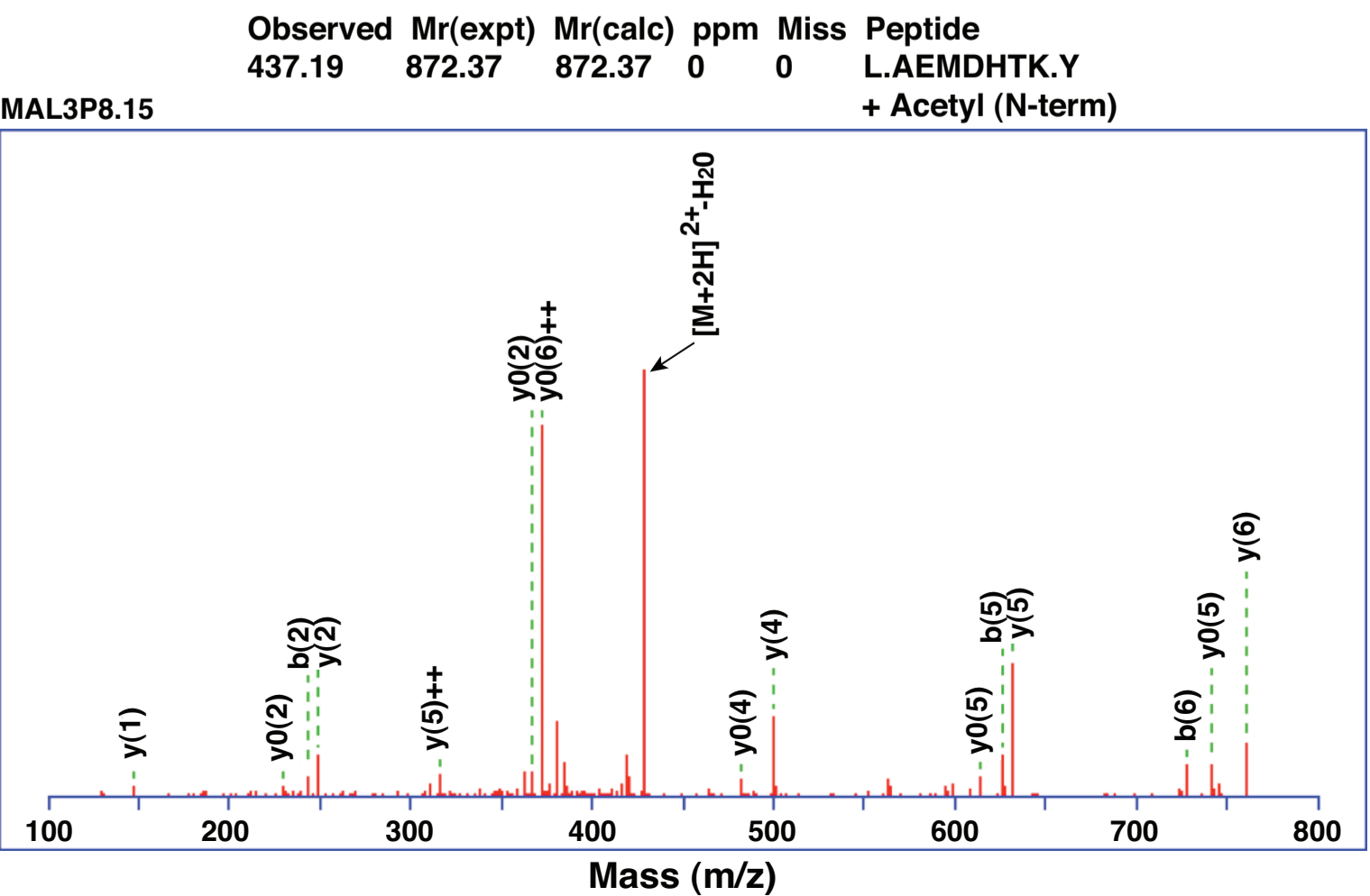

Supplement: Supplementary file 1 [file tra0010-0285-SD1.pdf]
