# Supplementary material for: Role of the Plasmodium Export Element in Trafficking Parasite Proteins to the Infected Erythrocyte
Source: Traffic. 2009 Jan 7;10(3):285–99. doi: 10.1111/j.1600-0854.2008.00864.x (PMC2682620; doi:10.1111/j.1600-0854.2008.00864.x)

# Fig. S2 Boddey et al.

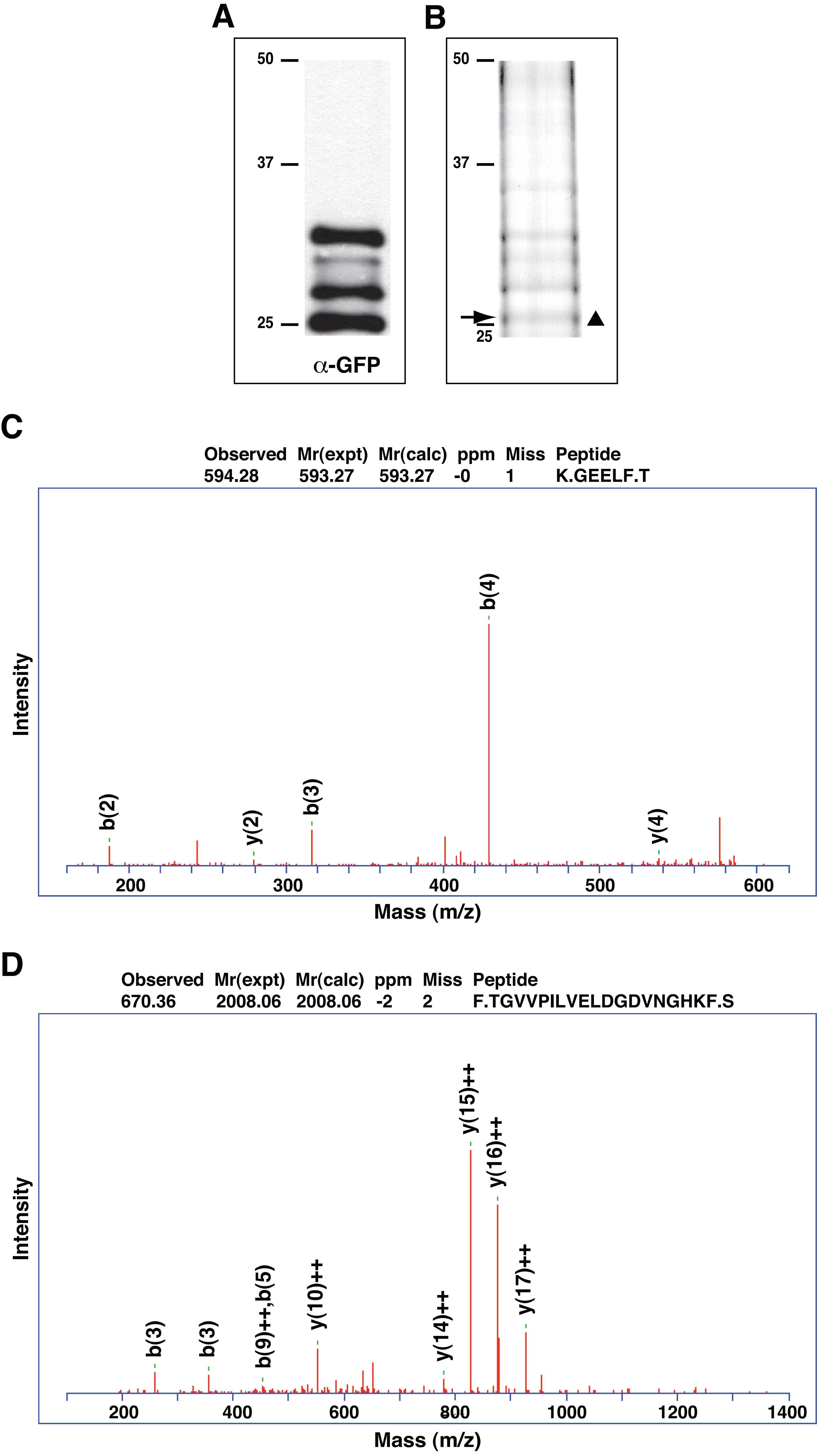

Supplement: Supplementary file 2 [file tra0010-0285-SD2.pdf]
